# Report of a Special Committee of the House of Assembly of the State of New-York, on the Present Quarantine Laws, 1846

**Published:** 1846-07

**Authors:** 


ART. VII.—*Report of a Special Committee of the House of Assembly of the State of New-York, on the present Quarantine Laws*, 1846.

The House of Assembly of 1845, appointed a committee of three to examine the Quarantine Laws of the port of New-York, to take testimony, report facts, and suggest such alterations as, in their opinion, should be deemed expedient, at the next session. The members of this committee were D. E. Wheeler, C. Comstock, and R. H. Hine. Accordingly, we had at the past session, a report of over three hundred octavo pages, embodying much interesting and useful information upon the several matters involved in the subject of Quarantine.

The committee drew up a series of interrogations, which they sent to several medical men of eminence, requesting their opinions in reply. These were generally given, with the reasons therefor, *in extenso*, and as opposing views are represented in these replies, the report is a valuable medical document, containing the most prominent arguments *pro.* and *con.* with reference to certain questions still in controversy. The chief points connected with the subject of Quarantine, relate to the origin and propagation of yellow fever. Is it ever indigenous in New-York, or is it always an exotic? If liable to importation, in what manner is it brought and communicated—by contagion, by infection, or by the elements of the morbid material introduced, and undergoing their peculiar combinations after their arrival? The reader will find different opinions, and a variety of facts bearing on these points in the report referred to. But, unfortunately, the questions remain precisely as they did before. No new light is shed upon the subject; and it will probably continue for some time to come, as it has for a long time past, one on which much is to be said in behalf of opposite doctrines.

The committee are entitled to great credit for the exertions which they appear to have made in the investigation of the subject; but it was not to have been expected that the medical testimony adduced would be sufficient to settle the vexed questions upon which different members of the profession are at issue. The gentlemen whose opinions were solicited, are, all, more or less distinguished, but it does not follow that they have all given to these particular subjects that degree of close investigation which entitles their opinions to peculiar weight. In so far as the testimony of opinion is concerned, the number of witnesses is too few to authorise deductions as to the predominent sentiments among the eminent portion of the profession. The views of the ten to whom the interrogatories were addressed, are by no means uniform. Taking the two first questions, which are the most important as affecting Quarantine regulations, the first is as follows: "Is yellow fever of domestic origin at the port of New-York?" The following are the names of those who answer the inquiry in the affirmative: Thomas Harris, M. D., U. S. Navy; Wm. Sweetser, M. D.; Wm. P. Hart, M. D.; W. S. W. Ruschenberger, M. D., U. S. Navy; D. Meredith Reese, M. D. In the negative are the following: Alex. F. Vache, M. D.; P. S. Townsend, M. D.; John W. Francis, M. D.; J. R. Manley, M. D.; Wm. C. Wallace, M. D. Provided this were the last tribunal of appeal, we might emphatically exclaim, 'who shall decide when doctors disagree,' since the number on each side is precisely the same! The second question is: "Is yellow fever imported by sea into that port?" On the affirmative, are Drs. Vache, Harris, Townsend, Francis, Sweetser, Manley, Wallace and Ruschenberger. On the negative, are Drs. Hart and Reese. The opinions are severally expressed with certain qualifications, &c., which we have not space to analyze, but must refer the reader to the report itself.

If it were deemed advisable to obtain as complete an investigation of the subject involved in the Quarantine question as facts, and the state of science will allow, the true method is, as Dr. Ruschenberger suggests, to entrust it to a medical commission composed of members selected for their professional fitness for such a duty. This commission should have ample time, and every facility, and the members should be properly compensated for their labor. We should be glad to see such an action taken by our legislature.

The committee, in the bill which accompanies their report, make some modifications of the existing Quarantine Laws, consisting chiefly in giving to the health officer a larger discretionary power, his actions subject to an appeal to a board consisting of the Mayor of New-York, the resident physician, and a practitioner designated by the Chamber of Commerce. This seems to us a judicious improvement. Indeed, we can see no other proper course to pursue, if it be deemed advisable to retain any Quarantine restrictions whatever, than to leave it to the judgment of an intelligent medical officer to decide as to the period of detention necessary, and the conditions of removing restraint. No specific regulations for general application can fail to be needlessly burthensome in very many particular instances. Should this alteration be adopted, not a few of the inconveniences attending Quarantine regulations would probably be obviated.

